# Approach to patients with diabetes and obesity in primary care

**DOI:** 10.1016/j.aprim.2023.102807

**Published:** 2023-11-14

**Authors:** Antoni Hormigo Pozoo, Desireé Torres Ortega, Antonio J. García Ruiz, José Escribano Serrano, María Escribano Cobalea, Nuria García-Agua Soler

**Affiliations:** a Unidad de Gestión Clínica San Andres-Torcal Clinical, Distrito Málaga-Guadalhorce, Málaga, Spain; b Programa de Doctorado en Biomedicina, Investigación Traslacional, y Nuevas Tecnologías en Salud, Facultad de Medicina, Universidad de Málaga, Málaga, Spain; c Departamento de Farmacología, Facultad de Medicina, Universidad de Málaga, Málaga, Spain; d IBIMA (Instituto de Investigación Biomédica de Malaga), Málaga, Spain; e Unidad de Gestión Clínica San Roque, Área de Gestión Sanitaria Campo de Gibraltar Este, San Roque, Cádiz, Spain; f Hospital Punta de Europa, Área sanitaria Campo de Gibraltar Oeste, Algeciras, Cádiz, Spain

**Keywords:** Weight control, Obesity, Type 2 diabetes, SGLT2 inhibitor, GLP-1, Primary care, Control de peso, Obesidad, Diabetes tipo 2, Inhibidor de SGLT2, GLP1, Atención primaria

## Abstract

**Aims:**

The aim of this study is to analyse the effect of pharmacological and non-pharmacological treatment on weight control in patients with diabetes and obesity.

**Design:**

Epidemiological, descriptive, cross-sectional study.

**Site:**

Primary care. In 11 health centres in Málaga and Cádiz during April and October 2022.

**Participants:**

281 patients over 18 years old with type 2 diabetes and obesity are included.

**Main measurements:**

Socio-demographics, clinical, treatment and lifestyle habits variables were obtained from medical records and personal interview. Descriptive statistics were obtained for continuous variables. Statistical tests were performed based on the nature of the variables.

**Results:**

Variables like marital status, level of education and occupation, and smoking habit, shows differences regarding the sex (*p* < 0.05). 82.3% of those who received education lost weight, compared to 67.5% of lost weight who received no health education (*p* = 0.004). GLP1 and SGLT2 were more commonly prescribed for women (*p* = 0.048), and SGLT2 more commonly prescribed for men (*p* = 0.047). Patients taking GLP1, SGLT2 or both, regardless of sex, weight loss during the study period was −3.1 kg (SE: 0.60), while the loss of those who took other medications was −1.33 kg (SE: 0.62). The mean difference was 1.75 kg (*p* = 0.046).

**Conclusions:**

In terms of weight loss, obese diabetics who took GLP1, SGLT2 or both were 2.5 times more likely to lose weight than those who did not. Healthy lifestyle choices are key to weight loss in obese diabetic patients.

## Abstract (es)

**Objetivo:**

Analizar el efecto del tratamiento farmacológico y no farmacológico en el control del peso en pacientes diabéticos obesos.

**Diseño:**

Estudio epidemiológico, descriptivo y transversal.

**Emplazamiento:**

Atención primaria: 11 centros de salud en Málaga y Cádiz durante abril y octubre de 2022.

**Participantes:**

Fueron incluidos 281 pacientes mayores de 18 años con diabetes tipo 2 y obesidad.

**Mediciones principales:**

Se obtuvieron variables sociodemográficas, clínicas, de tratamiento y de hábitos de vida a partir de registros médicos de entrevistas personales. Se obtuvieron estadísticos descriptivos para variables continuas. Se realizaron pruebas estadísticas en función de la naturaleza de las variables.

**Resultados:**

Variables como el estado civil, el nivel educativo, la ocupación y el hábito de fumar mostraron diferencias dependiendo del sexo (p < 0,05). El 82,3% de los que recibieron educación sobre salud perdieron peso en comparación con el 67,5% de los que perdieron peso y no la recibieron (p = 0,004). Los medicamentos GLP1 y SGLT2 se recetaron con mayor frecuencia a las mujeres (p = 0,048), mientras que los SGLT2 se prescribieron más en hombres (p = 0,047). Los pacientes que tomaron GLP1, SGLT2 o ambos, independientemente del sexo, perdieron 3,1 kg durante el estudio (EE: 0,60), mientras que aquellos que tomaron otros medicamentos perdieron 1,33 kg (EE: 0,62). La diferencia media fue de 1,75 kg (p = 0,046).

**Conclusiones:**

Los diabéticos obesos que tomaron GLP1, SGLT2 o ambos tenían 2,5 veces más probabilidades de perder peso que aquellos que no los tomaron. Un estilo de vida saludable es fundamental para la pérdida de peso en pacientes diabéticos obesos.

## Introduction

Type 2 diabetes is a metabolic disease characterised by hyperglycaemia and the development of microvascular and macrovascular complications. In addition, a large proportion of those affected are obese.^1^ The age and sex-adjusted incidence in Spain is 11.6/1000 people per year. The most common is type 2 diabetes, which is associated with obesity in 45% of cases (SIDIAP study).^2^, ^3^

The first therapeutic measure recommended by national and international guidelines is to make lifestyle changes, including a healthy diet, weight control, physical exercise, not smoking, and emotional well-being, all of which are essential in diabetes management.^4^ Therapeutic measures to treat diabetes also include pharmacological interventions to control blood glucose levels and help obese people lose weight. Molecules with proven efficacy should also be recommended.^5^

A recent study looked at the impact a healthy lifestyle had on people with diabetes. The results were low and did not specifically address clinical situations such as obesity which require increased enforcement of these recommendations.^6^ Pharmacological interventions by healthcare professionals (CAPTURE study) also suggest that there is room for improvement in the use of drugs with cardiovascular and weight-loss benefits.^7^

The aim of this study is to analyse the effect of pharmacological and non-pharmacological treatment on weight control in patients with diabetes and obesity.

## Methods

### Settings

An epidemiological, descriptive, cross-sectional, multicentre, regional study was conducted under routine clinical practice conditions.

Twenty physicians from 11 health centres in the provinces of Malaga and Cadiz participated. The inclusion criteria were: over 18 years of age; diagnosis of type 2 diabetes and obesity (BMI > 30), and having visited their primary care physician at least once in the last five years. All participants provided written informed consent.

Patients were recruited by consecutive sampling of obese type 2 diabetics who visited a doctor for any reason between April and October 2022. Data were collected from their medical records and personal interviews. In order to carry out the assessment, we required a blood test and weight control of the patient that had been taken within the last 6 months, and at least another for a period of no less than 1 year and no more than the previous 5 years to be able to make the comparison.

Patients attended a single visit which included an interview to obtain information not recorded in their medical history and to assess their current weight, lifestyle habits, exercise, treatment adherence, current pharmacological treatment, and its compliance with current guidelines. Patients were informed that their data were confidential.

To enable data collection and statistical analysis, clinical and socio-demographic variables were included in a specially designed online questionnaire that respected the anonymity and confidentiality of the individuals.

To calculate the sample size, we estimated population of approximately 30,000 people, in which 2671 people were diagnosed with diabetes (prevalence 8.903%) and in which 1200 people were estimated to have a BMI > 30. The required sample size was 280 participants (GRANMO 7.12 calculator, Institut Municipal d’Investigació Mèdica, Barcelona, Spain), 95% confidence level, 5% precision, 10% expected loss.

### Patient assessments

Available data on the following variables were collected from the medical records: Socio-demographics: age, sex, duration of DM, level of education, employment status, marital status. Healthy lifestyle habits: Physical activity. In order to measure adherence to exercise, we asked patients the question: “Do you do at least 30 min of moderate physical activity (e.g., brisk walking) five or more days per week?”. This allowed us to identify patients who were following the recommendations and those who were not.^8^

Emotional well-being. Three questions were asked regarding the emotional aspects of the IMEVID questionnaire.^9^

Clinical: weight, height, BMI, waist circumference, blood pressure, basal glycaemia, glycosylated haemoglobin, lipid profile, toxic habits (tobacco consumption), diabetes complications, and associated comorbidities. Patients were asked where they received follow-up care (primary/specialist, public/private) and whether they had received any type of health education. It is carried out by nurses in individual or group interventions, with indications are given to modify lifestyles (nutrition and exercise), through the supports that the health system provides us. The main dependent variable was weight loss, with a recommended weight loss of at least 5% for diabetes control.^5^

Treatment used: type of hypoglycaemic treatment, polymedication (more than 6 drugs), treatment adherence using the Haynes–Sackett test,^10^ and the adequacy of pharmacological treatment (according to the current recommendations of the guidelines and answered by the treating physician).

### Statistical methods

Analysis was performed with SPSS Statistics 26.0 software licensed to the University of Malaga (IBM Corp. Released 2019. IBM SPSS Statistics for Windows, Version 26.0. Armonk, NY: IBM Corp).

Descriptive statistics were obtained for continuous variables: mean and standard deviation or standard error (SD/SE). For qualitative variables: absolute and relative frequencies.

Statistical tests were performed based on the nature of the variables. Chi-squared test (Fisher's exact test when required) was used to examine the relationship between categorical variables. Student's *t*-tests or ANOVA were used for tests with quantitative variables when normality was met, otherwise *U*-Mann–Whitney and Kruskal–Wallis were used. A significance level of *α* = 0.05 was used for all statistical tests.

*Logistic regression analysis:* Logistic regression models allow us to analyse the results in both explanatory and predictive terms. We can determine the strength of association by means of the odds ratios of the predictor variables with the effect studied independently and determine the predictive value of each of them or of the model as a whole.^11^, ^12^

In our study, we analysed which model best fits the qualitative dependent variable (weight loss – yes/no) using the logistic regression method based on data from other known quantitative or qualitative variables acting as explanatory variables. The results of the independent variables are usually presented as odds ratios (OR) with 95% confidence intervals (CI).

The study was approved by the Cadiz Research Ethics Committee (PEIBA code 1125-N-22, reg.: 74.22).

**Study flowchart:** Epidemiological, descriptive, cross-sectional, multicentre, regional study to analyse the effects of pharmacological and non-pharmacological treatment on weight control in patients with diabetes and obesity. 

## Results

### Socio-demographic and clinical variables, and healthy lifestyle habits

281 patients were included with a mean age of 67.34 years (SD ± 10.46 years) and a mean diabetes duration of 10.30 years (SD ± 7.29 years). 55% of the patients were male, 73.3% were married, 43.8% had completed primary education and only 26% could read and write. In addition, 54.8% had retired and only 21.4% were employed. 83.3% of patients were followed up in primary care, 3.5% in specialist care, and 13.2% in both, and 76.2% had received individual or group education. Regular physical activity was reported by 39.1%, 87.6% were considered non-smokers (60.1% non-smokers and 27.4% former smokers) and 84.7% of patients reported acceptable emotional well-being.

The mean weight of the patients was 90.81 ± 15.32 kg. The mean height was 163.56 ± 9.73 cm. Waist circumference was 113.78 ± 10.44 cm. BMI was 33.85 ± 4.71. There were statistically significant differences (*p* < 0.005) between men and women in all variables, except waist circumference. The socio-demographic and clinical characteristics of the patients included in the study are shown in Table 1. Regarding the sex of the patients, we found statistically significant differences (*p* < 0.05) in the following variables: marital status, level of education and occupation, and smoking habit, especially in former smokers.

**Table 1:** Obese diabetic patients: sociodemographic and clinical variables.

Variable	Value	Female	Male	*N* (%)	*p* value
Gender		45.2%	54.8%	281 (100%)	

Age	<40 y	1.6%	0.6%	3 (1.1%)	0.651
	40–64 y	37.0%	37.7%	105 (37.4%)	
	65–75 y	35.4%	37.0%	102 (36.3%)	
	>75 y	26.0%	24.7%	71 (25.3%)	

Marital status	Single	5.5%	5.8%	16 (5.7%)	0.000
	Married	60.6%	83.8%	206 (73.3%)	
	Separated/divorced	28.3%	4.5%	43 (15.3%)	
	Widowed	5.5%	5.8%	16 (5.7%)	

Level of education	Can read/write	39.4%	16.2%	75 (26.%)	0.000
	Primary education	44.1%	43.5%	123 (43.8%)	
	Secondary education	11.8%	28.6%	59 (21.0%)	
	University education	4.7%	11.7%	24 (8.5%)	

Occupation	Active worker	16.5%	25.3%	60 (21.4%)	0.000
	Unemployed	5.5%	6.5%	17 (6.0%)	
	Household	39.4%	–	50 (17.8%)	
	Pensioner/retired	38.6%	68.2%	154 (54.8%)	

DM developments	<10 years	60.6%	55.8%	163 (58.0%)	0.419
	≥10 years	39.4%	44.2%	118 (42.0%)	

Therapeutic education	One to one	74.8%	76.6%	213 (75.8%)	0.529
	Grupal	0.8%	–	1 (0.4%)	
	None	24.4%	23.4%	67 (23.8%)	

Treatment pattern	Monotherapy	36.2%	29.9%	92 (32.7%)	0.305
	Doble oral	30.7%	25.3%	78 (27.8%)	
	Triple oral	16.5%	19.5%	51 (18.1%)	
	Basal insulin + ADO	5.5%	9.1%	21 (7.5%)	
	Basal Insulin + aGLP1	0.8%	–	1 (0.4%)	
	Basal insulin + aGLP1 + ADO	9.4%	12.3%	31 (11.0%)	
	Basal + prandial insulin	0.8%	1.3%	3 (1.1%)	
	Basal + prandial insulin + ADO	–	2.6%	4 (1.4%)	

Polymedicated	Yes	63.8%	53.2%	163 (58.0%)	0.075
	Non	36.2%	46.8%	118 (42.0%)	

The clinical variables of the patients included in the study are analysed in Table 2. Regarding the patients’ medical history (comorbidities), statistically significant differences were found only for neuropathies and ischaemic heart disease—more frequent in men—and anxiety/depression—more frequent in women.

**Table 2:** Obese diabetic patients: clinical variables.

	*N*	Median	IQ rank	Shapiro–Wilk test	*p*-Value	Mean	SD	95% CI		*p*-Value
								Lower	Upper	*U*-Mann–Whitney
*Age (in years)*										
Male	154	68.00	16.75	0.978	0.014	66.97	10.4	65.32	68.63	0.627
Female	127	68.00	16.00	0.988	0.307	67.79	10.6	65.93	69.65	
Total	281					67.34	10.5	66.11	68.57	

*Years of diabetes evolution*										
Male	154	10.00	10.75	0.946	<.001	10.44	6.71	9.37	11.5	0.318
Female	127	8.00	10.00	0.869	<.001	10.13	7.95	8.73	11.52	
Mean	281					10.3	7.29	9.44	11.15	

*Systolic BP*										
Male	154	13,000	10.00	0.947	<.001	13,364	12.1	131.7	135.6	0.295
Female	127	13,000	17.00	0.973	0.012	13,205	11.6	130	134.1	
Total	281					13,292	11.9	131.5	134.3	

*Diastolic BP*										
Male	154	76.00	10.00	0.963	<.001	76.53	10.1	74.92	78.14	0.298
Female	127	80.00	13.00	0.962	0.001	77.64	9.19	76.02	79.25	
Total	281					77.03	9.7	75.89	78.17	

*Basal blood glucose*										
Male	154	12,500	35.00	0.813	<.001	13,394	41.1	127.4	140.5	0.746
Female	127	12,800	39.00	0.916	<.001	133.3	37.2	126.8	139.8	
	281					13,365	39.3	129	138.3	

*Cholesterol*										
Male	154	15,600	60.00	0.979	0.021	15,981	37.3	153.9	165.7	<0.001
Female	127	18,100	54.50	0.969	0.005	18,351	37.2	177	190	
Total	281					17,052	38	165.9	175.1	

*HDL cholesterol*										
Male	154	39.00	10.50	0.98	0.026	39.14	7.98	37.87	40.41	<0.001
Female	127	46.00	14.50	0.975	0.02	47.09	10.4	45.26	48.92	
Total	281					42.73	9.97	41.56	43.9	

*LDL cholesterol*										
Male	154	88.00	48.75	0.924	<.001	93.91	36.3	88.12	99.69	0.011
Female	127	99.00	41.00	0.954	<.001	103.3	32.5	97.6	109	
Total	281					98.15	34.9	94.05	102.3	

*Triglycerides*										
Male	154	14,950	96.75	0.916	<.001	16,327	74.3	151.4	175.1	0.412
Female	127	15,400	91.00	0.895	<.001	16,952	76	156.2	182.9	
Total	281					16,609	75	157.3	174.9	

*Glomerular filtration rate*										
Male	154	80.00	27.00	0.891	<.001	73.82	18.2	70.94	76.71	0.412
Female	127	78.00	24.00	0.959	<.001	76.07	17.6	72.98	79.16	
Total	281					74.84	17.9	72.74	76.94	

*Weight (kg)*										
Male	154	92.50	14.30	0.902	<.001	953,026	14.5	93	97.61	<0.001
Female	127	82.50	16.50	0.908	<.001	853,709	14.6	82.81	87.93	
Total	281					908,139	15.3	89.01	92.61	

*Size (cm)*										
Male	154	17,000	10.00	0.988	0.199	169,695	7.48	168.5	170.9	<0.001
Female	127	15,600	10.00	0.935	<.001	156,138	6.41	155	157.3	
Total	281					163,568	9.73	162.4	164.7	

*Body mass index*										
Male	154	32.02	3.31	0.864	<.001	329,939	3.88	32.38	33.61	<0.001
Female	127	34.21	6.29	0.916	<.001	348,783	5.38	33.93	35.82	
Total	281					338,456	4.71	33.29	34.4	

*Waist circumference (cm)*										
Male	154	11,450	11.00	0.968	0.001	114,721	9.49	113.2	116.2	0.026
Female	127	11,100	13.50	0.916	<.001	112,638	11.4	110.6	114.6	
Total	281					113,779	10.4	112.6	115	

*HbA1c (%)*										
Male	154	6.85	12.00	0.894	<.001	70,649	1.17	6.88	7.25	0.44
Female	127	6.70	12.00	0.872	<.001	69,672	1.15	6.77	7.17	
Total	281					70,208	1.16	6.88	7.16	

### Treatment variables

Most patients control their disease with monotherapy (32.7%) followed by dual and triple therapy (27.8% and 18.1%, respectively). Other patients (21.4%) haven’t got pharmacological treatment, only a diet prescribed. 54.4% of obese diabetics are treated with GLP1 (8.9%) or SGLT2 (27%) or both (18.5%). There were significant differences regarding patients’ sex and the use of GLP1 and SGLT2, with GLP1 and SGLT2 more commonly prescribed for women (*p* = 0.048), and SGLT2 more commonly prescribed for men (*p* = 0.047).

Fifty-eight percent of patients were polymedicated and 90% reported good adherence. 68.7% of the physicians believed the treatment provided was in line with the current recommendations.

No statistically significant differences were found between the sex of the patient and disease progression, therapeutic education received, pattern of antidiabetic medication used, or polymedication (Table 1).

**Figure 1:**
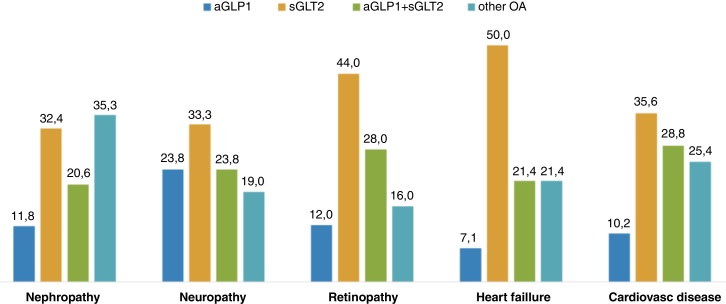
Bivariate analysis: clinical history and treatment.

Regarding the types of treatment and some of the patients’ clinical histories, there were statistically significant differences (Pearson's Chi-squared, *p* < 0.05) in the type of treatment in all patients analysed, as shown in Fig. 1.

### Weight loss

The mean weight loss of the obese diabetic patients studied over the past year was 2.32 kg (95%CI: 1.46–3.17). 35.9% of patients (*n* = 101) gained weight (mean ± SE: 3.29 ± 0.34 kg), and 57.7% of patients (*n* = 162) lost weight (mean ± SE: 6.31 ± 0.52 kg). Only 6.4% of patients (*n* = 18) maintained their weight—5 women and 13 men (3.9% and 8.4%).

We found that according to the sex of the patients and weight control, 32.3% (*n* = 41) of the women gained weight, on average 3.38 kg, and 63.8% (*n* = 81) lost weight, on average −6.88 kg (SE: 0.798). In men, 38.9% (*n* = 6) gained an average of 4.10 kg, and 52.6% lost an average of −5.91 kg (*n* = 81). No statistically significant differences were found (*p* = 0.349). Furthermore, weight loss was less than 2% in 39.6% of the patients (36.2% women, 47.4% men) and more than 2% in 57.6% (63.8% women, 52.6% men) (*p* = 0.038).

Mean weight loss with GLP1 or SGLT2 or both was 3.74 kg, compared to 1.65 kg with other ADOs. There were statistically significant differences (*p* = 0.039). Of all the patients analysed (*n* = 281), 35.9% did not lose weight (*n* = 101). 17.4% maintained their weight or lost less than 2% (*n* = 49). 19.2% of patients lost between 2% and 5% (*n* = 54). A further 27.4% lost more than 5% of their weight compared to the previous year (*n* = 77). Of all patients who lost more than 5% of their baseline weight, 64.9% were taking GLP1 or SGLT2 or GLP1 + SGLT2 (10.4%, 24.7% and 29.9%, respectively) and 35.1% were taking other ADOs.

Regarding the level of education, no statistically significant differences were found between the patients’ weight losses, although a trend was observed, so that the differences between a university education and no education were statistically significant (*p* < 0.05).

With regard to the health education offered to these patients, it should be noted that 67.5% of those who received no education (*n* = 67) lost weight, while 82.3% of those who received education lost weight, and these differences were statistically significant (*p* = 0.004).

In patients taking appropriate medications (GLP1, SGLT2 or both), regardless of patient sex, weight loss during the study period was −3.1 kg (SE: 0.60), while those taking other medications experienced a weight loss of −1.33 kg (SE: 0.62). There were statistically significant differences (*p* = 0.046). The mean difference was 1.75 kg.

When comparing weight loss (yes/no) with taking or not taking GLP1 and SGLT2 drugs, statistically significant differences were found, *p* = 0.009 (Fisher's exact test). 59.3% of patients who lost weight were prescribed GLP1, SGLT2 or both, while 40.7% were prescribed other treatments.

### Logistic regression analysis

Table 3 shows the statistics for the variables included in each model.

**Table 3:** Descriptive factors of the logistic regression model.

Any weight loss (yes/no)	*p* value (sig.)	OR (Exp(*B*))	95%CI (exp *B*)	
			Lower	Upper
*Sex*	0.042	1.677	1.020	2.758
*Health education*	0.002	2.416	1.364	4.278
*aGLP1* *+* *sGLT2*	0.009	2.495	1.260	4.940

*Weight loss of more than 5% (yes/no)*				
Health education	0.010	2.634	1.256	5.527
aGLP1 + sGLT2	0.005	2.505	1.260	4.721

### Dependent variable

Any weight loss (yes/no): The model classified patients who DO lose weight with an accuracy rate of 92.4%. It included the following variables: sex, health education, and medication (GLP1 + SGLT2). Thus: Women are 1.677 times more likely to lose weight than men. Patients who received health education were 2.416 times more likely to lose weight than those who did not. Subjects whose treatment included GLP1 + SGLT2 were 2.495 times more likely to lose weight than those not taking these drugs. Female subjects who receive health education at their health centre and take GLP1 + SGLT2, are 83% more likely to lose weight, while those who are male, do not receive health education and do not take the medication are 32% more likely to lose weight.

### Dependent variable

Weight loss greater than 5% (yes/no): The model classified patients who did NOT lose 5% or more weight with an accuracy rate of 100%. It included the following variables: health education and taking both medications (GLP1 + SGLT2). Thus: Patients who received health education were 2.634 times more likely to lose more than 5% more weight than those who did not. Subjects whose treatment included GLP1 + SGLT2 are 2.505 times more likely to lose more than 5% of their body weight than those not taking these drugs.

## Discussion

The aim of the study was to determine the efficacy of the interventions carried out by a public service (Andalusian Health Service) in obese diabetic patients over a five-year period, in terms of their impact on weight and the follow-up by health professionals of the pharmacological recommendations made by the diabetes care process in Andalusia,^13^ and by national and international guidelines.^5^, ^13^ As such, the interest of this study stems from the lack of data assessing the effectiveness of healthcare professionals’ actions on weight.

In our study, 76.2% had received health education, 39.1% were physically active, 87.5% did not smoke, 90% adhered to the prescribed treatment, and 84.7% considered their emotional well-being satisfactory. We find similar data if we compare our study with other results obtained in Spain,^14^ where 88.3% had received individual or group therapeutic education, 45.8% were physically active, 87.6% were non-smokers, 62.8% considered their emotional well-being satisfactory and just over 20% of patients followed a Mediterranean diet. The NHANES III survey^15^ in the USA assessed weight, exercise, tobacco use, and fruit and vegetable intake—the data found were similar to those of the Spanish study.^14^ Both studies were conducted in a diabetic population aged > 18 years in which less than 50% of the sample was obese.

It is noteworthy that only 27% of patients have managed to reduce their weight by at least 5% in the last five years, which is recommended for diabetes control. However, if we look at a weight loss of >2%, we see that 47% of patients have achieved this.

In terms of treatment, GLP1 and SGLT2 are more commonly prescribed in women and SGLT2 in men, and even more so in people over 75 years of age, possibly because of the higher prevalence of ischaemic heart disease and the relationship between heart failure and age. According to the recommendations of national and international guidelines, the adequacy of treatment in people with diabetes and obesity is 54.4%, while 68.7% of health professionals consider treatment to be adequate. Regarding the comorbidities associated with diabetes and obesity, the adequacy of treatment for cardiovascular disease according to guidelines is 71.2% a figure much higher than that found in the CAPTURE study,^7^ which was 21.5%.

Treatment with guideline-recommended weight-loss drugs is adequate in just over half of patients, and in patients with associated cardiovascular disease this figure is 71.4%, much higher than in previous studies.

About the limitations of the study, the patients were selected from a population receiving care by means of non-stratified consecutive sampling, with the consequent selection bias. Researchers took great care in collecting the data, although we cannot rule out the possibility that respondents may have been less than truthful in answering the questions posed. It is a cross-sectional observational study, suitable for understanding changes in lifestyle and weight, pharmacological treatment, its adaptation to national and international guidelines, and the clinical characteristics of obese diabetic patients seen in primary care in Spain, but it is not applicable to other populations or health systems.

## Conclusion

In terms of weight loss, obese diabetics who took GLP1, SGLT2 or both were 2.5 times more likely to lose weight than those who did not. Healthy lifestyle choices are key to weight loss in obese diabetic patients.

## Ethical considerations

The study was approved by the Cadiz Research Ethics Committee (PEIBA code 1125-N-22, reg.: 74.22). All participants signed the informed consent.

## Conflict of interest

All authors declare that they have no conflicts of interest.
